# Overlapping Transmission Networks of Early Syphilis and/or Newly HIV Diagnosed Gay, Bisexual and Other Men Who Have Sex with Men (MSM): Opportunities for Optimizing Public Health Interventions

**DOI:** 10.1007/s10461-020-02840-2

**Published:** 2020-04-01

**Authors:** Jacky M. Jennings, Carla Tilchin, Benjamin Meza, Christina Schumacher, Errol Fields, Carl Latkin, Anne Rompalo, Adena Greenbaum, Khalil G. Ghanem

**Affiliations:** 1 Center for Child and Community Health Research (CCHR), Department of Pediatrics, Johns Hopkins School of Medicine, Baltimore, MD USA; 2 Department of General Internal Medicine, Johns Hopkins School of Medicine, Baltimore, MD USA; 3 Department of Health, Behavior and Society, Johns Hopkins Bloomberg School of Public Health, Baltimore, MD USA; 4 STI/HIV Program, Baltimore City Health Department, Baltimore, MD USA; 5 Department of Infectious Disease, Johns Hopkins School of Medicine, Baltimore, MD USA; 6 Department of Pediatrics, School of Medicine, The Johns Hopkins University Bayview Medical Center, 5200 Eastern Avenue, Mason F. Lord Bldg-Center Towers, Suite 4200, Baltimore, MD 21224 USA

**Keywords:** MSM, Syphilis, HIV, Sex partner meeting places, Venue co-affiliation networks

## Abstract

Syphilis and HIV among gay, bisexual and other men who have sex with men (MSM) are syndemic suggesting current prevention strategies are not effective. Sex partner meeting places and their networks may yield effective and optimal interventions. From 2009 to 2017, 57 unique venues were reported by > 1 MSM and 7.0% (n = 4), 21.1% (n = 12) and 71.9% (n = 41) were classified as syphilis, HIV or co-diagnosed venues, respectively. Forty-nine venues were connected in one main network component with four online, co-diagnosis venues representing 51.6% of reports and the highest degree and eigenvector centralities. In a sub-analysis during a local syphilis epidemic, the proportion of venues connected in the main component increased 38.7% (61.5% to 86.4%); suggesting increasing overlap in syphilis and HIV transmission and density of the venue network structure over time. This network analysis may identify the optimal set of venues for tailored interventions. It also suggests increasing difficulty of interrupting network transmission through fragmentation.

## Resumen

La sífilis y el VIH entre hombres homosexuales, bisexuales y otros que tienen relaciones sexuales con hombres (HSH) son sindémicos, lo que sugieren que las estrategias de prevención actuales no son efectivas. Los lugares de encuentro de parejas sexuales y sus redes pueden producir intervenciones efectivas y óptimas. De 2009 a 2017, 57 lugares únicos fueron reportados por > 1 HSH y 7.0% (n = 4), 21.1% (n = 12) y 71.9% (n = 41) fueron clasificados como sífilis, VIH o lugares co-diagnosticados, respectivamente. Cuarenta y nueve lugares se conectaron en un componente principal de la red con cuatro lugares de co-diagnóstico en línea, que representan 51.6% de los informes y el más alto grado y centralidades de vector propio. En un subanálisis durante una epidemia de sífilis local, la proporción de lugares conectados en el componente principal aumentó 38.7% (61.5% a 86.4%); lo que sugiere una mayor superposición en la transmisión de sífilis y VIH y la densidad de la estructura de la red de lugares a lo largo del tiempo. Este análisis de red puede identificar el conjunto óptimo de lugares para intervenciones a medida. También sugiere una creciente dificultad para interrumpir la transmisión de la red a través de la fragmentación.

## Introduction

The current syphilis epidemic in the United States (U.S.) among gay, bisexual and other men who have sex with men (MSM) is closely linked to the HIV epidemic. In 2017, MSM accounted for 79.6% of all primary and secondary (P&S) syphilis cases among males and 57.9% of all P&S syphilis cases overall [1]. Approximately half of MSM with P&S syphilis were also living with HIV [2]. This syndemic of HIV and syphilis is particularly acute for Black MSM. In 2017, the National HIV Behavioral Surveillance found that HIV prevalence among Black MSM was 39% among the 23 selected U.S. cities [3].

The syndemic is likely due in part to the fact that syphilis infection can facilitate the transmission and acquisition of HIV infection [4–6]. There is an estimated 2- to 5-fold increase in the risk of HIV acquisition among persons with syphilis [3, 7, 8]. In addition, syphilis may be an indicator of ongoing sexual risk behaviors and membership in a high transmission network for both syphilis and HIV. The syndemic nature of HIV and syphilis suggests a need to better understand the network transmission dynamics of syphilis and HIV among MSM and specifically, Black MSM.

Traditional methods of syphilis (and later, HIV) control were designed to address network transmission. Local health departments have traced the partners of patients with syphilis, a process called contact tracing, since the 1930s for the purpose of treatment and prevention [9]. While useful for disrupting network transmission, these methods have limitations for understanding transmission dynamics because missing links are common. Missing links may be due to casual or anonymous contacts with less reliable locating information [10], or mistrust of government institutions including the health department staff eliciting contact information [11]. Missing links may also be due to recency and recall bias, as demonstrated by inconsistent reporting of particular partners, self-reported forgetting and partners reported in some interview modes such as daily diaries and not in others [12–14]. It is therefore possible that contact tracing does not represent or even misrepresents the dynamics of the population and may miss high-risk transmission events and network connections [15–18].

An alternative or complement to contact tracing is to collect information on sex partner meeting places. Sex partner meeting places are venues such as a bar, club, internet application (app), website, market, park, school, or street corner where individuals meet sex partners forming sexual dyads and larger sexual networks. The rationale is that sex partner meeting places are less subject to the limitations of contact tracing and may be an effective means to access priority populations for interventions such as providing access to pre-exposure prophylaxis (PrEP), antiretroviral therapy (ART), testing and linkage to care/treatment for STIs and HIV, and messaging for undetectable equals untransmissable (U = U) campaigns and safer sex practices [15, 19, 20].

The use of specific sex partner meeting places to disrupt network transmission is not new. Bathhouses were an important meeting place to find anonymous sex partners among MSM in San Francisco in the 1980s and were used to create sexual networks in which the first HIV/AIDS cases were traced and mapped [19]. More recently, at least one outbreak of syphilis was linked to seeking sex partners through an online chatroom [21]. Venues where early syphilis and/or newly HIV diagnosed MSM report recently meeting their sex partners are likely to be access points for other members of a syphilis and/or HIV transmission network. In addition, if there is substantial transmission overlap, dual strategies for syphilis and HIV prevention and treatment may be optimal and more cost-effective than separate disease-specific strategies.

In our recent work, we have taken an innovative approach to analyzing the sex partner meeting places of newly HIV diagnosed MSM [22], an approach utilized by a few other researchers as well [23–25]. We have been approaching the study of venues like the study of networks by mapping the network connections of venues through venue affiliation and venue co-affiliation networks. This is made possible by the fact that some individuals report more than one venue, which creates connectivity between venues. Venue co-affiliation network analyses can reveal tightly connected venues where syphilis and/or HIV transmission may be occurring by evaluating metrics related to venue centrality (i.e. how many times the venue was nominated) and network density (i.e. how closely venues are connected to each other or how socially intertwined the venues are) [26, 27]. Measures of venue centrality and network density have been useful in studying HIV, STI, and tuberculosis transmission networks [28–31] and may be one approach to prioritizing venues for targeted control. Venue co-affiliation network analyses may lead to new control activities by explicitly recognizing and taking advantage of both the nominated venues themselves and the connections between venues (i.e. network structure), and optimizing activities across, for example, syphilis and HIV.

The overall goal of this work is to inform innovative, data-driven public health control strategies for reducing STIs and HIV. Among MSM with early syphilis and/or a new HIV diagnosis, the objectives are to (1) characterize the connectivity of sex partner meeting places to assess venue co-affiliation network structure and examine transmission overlap; (2) in a sub-analysis, describe temporal changes in the co-affiliation network structure over a 5 year period when early syphilis cases increased by 40% among MSM. We hypothesize that a majority of venues will be nominated by both early syphilis and/or newly HIV diagnosed individuals, and that a small number of venues with the greatest centrality measures will reveal a discrete set of venues that may be useful for public health control activities and/or interventions. We also hypothesize that the venue co-affiliation networks of early syphilis and/or newly HIV diagnosed MSM will become increasingly dense over the 5-year period during an increase in early syphilis cases among MSM.

## Methods

### Setting

Baltimore City, Maryland ranks among U.S. cities with the highest incidence of syphilis and HIV among MSM. In 2017, the rate of P&S syphilis was 3.6-fold higher than the national rate (34.2 vs. 9.5 per 100,000) [2]. Additionally, 69.6% (n = 268) of all reported early syphilis cases (P&S and early latent) were among MSM, among whom 82.2% (n = 220) were Black MSM (Unpublished results). From 2010 to 2014, early syphilis among MSM increased 40%, and this time period was selected for the sub-analysis in objective two [32]. In 2016, the Baltimore–Columbia–Towson metropolitan statistical area (MSA) ranked 14th in the nation for HIV prevalence and 8th in the nation for cumulative number of persons living with HIV ever classified as AIDS [33].

### Study Population

This work was conducted through a public health-academic partnership between the STI/HIV prevention and control program of the Baltimore City Health Department (BCHD) and the Johns Hopkins University Center for Child and Community Health Research (CCHR). Reporting of syphilis and HIV to state and local health departments is legally mandated in Maryland. The BCHD refers all early syphilis and/or newly HV diagnosed individuals with a primary residential address within Baltimore City for partner services. An early syphilis case is defined as an individual with primary, secondary, or early latent syphilis as staged by a clinical provider or a BCHD disease intervention specialist (DIS). A new HIV diagnosed case is defined as an individual without a prior record of an HIV infection reported in the U.S. During routine partner services, in addition to collecting demographic, risk behavior, and sex partner information, the BCHD routinely collects information on sex partner meeting places (e.g., name of bar, club, app, website, market, park, school, or street corner). The information is elicited by a question to the individual, “Where did you meet your sex partners in the past 12 months?”.

For this study, we used public health surveillance data from MSM with an early syphilis and/or new HIV diagnosis and reporting at least one sex partner meeting place from 2009 to 2017. Male cases were considered to be MSM if during the partner services interview, they self-identified as gay or bisexual or reported having sex with men. The recall for sexual risk behaviors (i.e. number of sex partners, commercial sex work) and drug use during partner services interviews was determined by the critical period for the diagnosis: 12 months for HIV diagnoses and 3, 6, and 12 months for primary, secondary, and early latent syphilis diagnoses, respectively. In this analysis, drug use was defined as self-report of injection drug use or any method of consumption of the following drugs—cocaine, heroin, and/or methamphetamines.

A specific sex partner meeting place was defined as a physical location that could be placed on a map or an online location that was a specific application or website. Reported places that were non-specific venues or regions, such as “bars,” “online” and “West Baltimore”, were not included. In order to build the venue co-affiliation networks, only venues reported by more than one individual were included in the analysis. This study used routine public health surveillance information and was considered exempt from human subjects research by the Johns Hopkins Medicine IRB.

### Statistical Analyses

#### Individual-Level Measures

Summary statistics were generated to describe and compare the study population overall and by diagnosis (early syphilis only, new HIV diagnosis only, and co-diagnosed with syphilis and HIV) using chi-squared tests or t-tests, as appropriate. Descriptive statistics included demographics (e.g. age, race), sexual risk behavior (e.g. number of sex partners), and number and type of venues reported. In addition, we compared individuals who reported a sex partner meeting place (i.e. the study population) to those who did not report a sex partner meeting place to identify potential biases in the study population.

#### Venue-Level Measures

Venues were classified by typology, diagnoses, and sexual risk behaviors. Venues were classified into six typologies: internet/app (i.e. online), bar/club, street/park/neighborhood, market/mall, or other [34]. Venues were also classified based on the diagnoses of the individuals reporting the venue. Venues were classified as a “syphilis venue” if only individuals diagnosed with only early syphilis nominated the venue. Venues were classified as an “HIV venue” if only individuals newly diagnosed with HIV nominated the venue. Venues were classified as an “syphilis and HIV venue” (i.e. co-diagnosis venue) through two definitions; (1) if both an individual with an early syphilis diagnosis and an individual with a new HIV diagnosis nominated the venue and/or (2) if at least one individual co-diagnosed with early syphilis and HIV, including individuals known to be HIV positive at the time of their early syphilis diagnosis, nominated the venue. Venues were also classified for some analyses as single diagnoses venues (i.e. syphilis venues and separately, HIV venues) and co-diagnosis venues (as above). Venues were also classified based on the individual level sexual risk behaviors reported by individuals (i.e. compositional venue characteristics) who also nominated the venue including commercial sex work, any drug use, anonymous sex and condomless sex.

#### Network-Level Measures

To describe the co-affiliation network, we calculated the proportion of venues by diagnoses and sexual risk behaviors (i.e. commercial sex work and drug use) of the individuals who nominated the venue and by venue typology.

Two measures of venue centrality, degree centrality and eigenvector centrality, were calculated to evaluate the relative prominence of specific venues in the venue co-affiliation network for objective one. Degree centrality was calculated as the number of ties (i.e. a tie is where two venues share a patron) the venue had to all other venues within the network [35]. A venue with a degree centrality of eight could have one tie each to eight unique venues, eight ties to one venue, or anything in between. Degree centrality measures the number of connections or potential transmission events a venue has in the network.

Eigenvector centrality was calculated as the number of ties the venue had within the network (i.e. degree centrality) weighted by the degree centrality of each of the venues’ adjacent venues in the network [35]. For example, a venue with three ties to three venues that have no other ties in the network has a lower eigenvector centrality than a venue with three ties to three venues that themselves have two additional ties each. When applied to transmission dynamics, venues with a higher eigenvector centrality represent higher risk venues because they are more highly connected to other venues that themselves are highly connected.

Two measures of network density, connectedness and compactness, were calculated to assess changes in the venue co-affiliation network density over time for objective two. Connectedness was calculated as the proportion of venue pairs that could reach each other by a path of any length to all possible venue pairs in the network (i.e. the proportion of nodes in the main component) [35]. A network with seven nodes in one main component and three unconnected nodes (i.e. isolates) has a connectedness of 0.7. Compactness was calculated as the proportion of venue pairs that can reach each other by a path of any length (i.e. connectedness) weighted inversely by the lengths of the paths connecting the nodes [35]. When applied to transmission dynamics, compactness takes into account that disease will spread more quickly within networks with shorter distances between nodes (i.e. denser).

#### Hypothesis Testing

To characterize the connectivity of sex partner meeting places, we created a venue co-affiliation network of sex partner meeting places reported by at least two individuals over the entire study time period (objective one) from 2009 to 2017 and in a sub analysis, by each year (objective two) from 2010 to 2014. Venues were connected in the network, i.e. shared a tie, when they shared at least one patron.

To determine syphilis and HIV transmission overlap (objective one), we first described the connectivity of syphilis only and HIV only venues (i.e. single diagnosis venues) vs. co-diagnosis venues and then we compared the median degree centrality and median eigenvector centrality of single diagnosis venues vs. co-diagnosis venues using Mann–Whitney tests (i.e. a nonparametric test of difference).

To assess changes in density and transmission overlap over time (objective two), we calculated the median degree centrality and median eigenvector centrality of the co-affiliation network for each year. The percent change in median degree centrality and median eigenvector centrality of the co-affiliation network in 2010 was compared to 2014 using Mann–Whitney tests. We also calculated two additional network measures, connectedness and compactness, by year to assess changes in network density over time. Changes in connectedness and compactness were described using percent changes from 2010 to 2014.

Descriptive analyses and network analyses were performed using Stata Version 14 and UCINET Version 6.657. Network visualizations were built in R version 3.5.0 using the iGraph package [36]. For all analyses, statistical significance was determined by a p-value of < 0.05.

## Results

### Study Population

Between January 2009 and December 2017, 6175 individuals diagnosed with early syphilis and/or a new HIV diagnosis were reported to the BCHD. Among these 6175 individuals, 77.1% (n = 4758) were males, among whom 61.4% (n = 2921) were MSM. Among MSM, 88.5% (n = 2585) received partner services interviews, of whom 24.8% (n = 641) reported at least one specific venue and were included in this analysis. MSM who reported a venue were not statistically different by age, race, ethnicity or diagnoses compared to MSM who did not report a venue (*data not shown*).

Among these 641 individuals, 31.8% (n = 204) were diagnosed with early syphilis only, 42.3% (n = 271) were diagnosed with HIV only, and 25.9% (n = 166) were co-diagnosed with early syphilis and new HIV (Table 1). Among those diagnosed with early syphilis only, 15.9% (n = 43) were diagnosed with primary syphilis, 43.2% (n = 117) were diagnosed with secondary syphilis, and 41.0% (n = 111) were diagnosed with early latent syphilis. Among those co-diagnosed with early syphilis and new HIV, 4.5% (n = 8) were diagnosed with primary syphilis, 30.7% (n = 51) were diagnosed with secondary syphilis, and 28.9% (n = 48) were diagnosed with early latent syphilis (*data not shown*).

**Table 1:** Characteristics of gay, bisexual and other men who have sex with men (MSM) with early syphilis and/or a new HIV diagnosis and who reported at least one specific venue stratified by diagnosis, Baltimore City, 2009–2017 (n = 641)

Characteristics	Overall (n = 641)	Early syphilis only diagnosis (n = 271)	HIV only diagnosis (n = 204)	Early syphilis and HIV co-diagnosis^a^ (n = 166)
Demographics				
Age, median (IQR)	27 (11.00)	26 (10.00)	25 (9.00)	31 (16.00)
Race				
Black, n (%)	507 (79.1)	216 (79.7)	160 (78.4)	131 (78.9)
White, n (%)	97 (15.1)	39 (14.4)	30 (14.7)	28 (16.9)
Other, n (%)	37 (5.8)	16 (5.9)	14 (6.9)	7 (4.2)
Ethnicity, Latino, n (%)	32 (5.0)	13 (4.8)	12 (5.9)	7 (4.2)
Sexual and drug behaviors				
Number of sex partners during critical period^b^ (n = 635), median (IQR)	2 (50.00)	2 (20.00)	2 (35.00)	2 (50.00)
Commercial sex work, n (%)	46 (7.2)	18 (6.6)	17 (8.3)	11 (6.6)
Any drug use, n (%)^c^	56 (8.7)	22 (8.1)	17 (8.3)	17 (10.2)
Anonymous sex, n (%)	345 (53.8)	154 (56.8)	106 (52.0)	85 (51.2)
Sex without a condom, n (%)	609 (95.0)	255 (94.1)	193 (94.6)	161 (97.0)
Venue-related information				
Total number of venue nominations	836	335 (40.1)	293 (35.0)	208 (24.9)
Median number of venue nominations (IQR)	1 (6.00)	1 (5.00)	1 (4.00)	1 (3.00)
Online venues, n (%)	622 (74.4)	253 (75.5)	232 (79.2)	137 (65.9)
Bars or clubs, n (%)	144 (17.2)	53 (15.8)	46 (15.7)	45 (21.6)
Streets, parks or neighborhoods, n (%)	33 (4.0)	15 (4.5)	7 (2.4)	11 (5.3)
Markets or malls, n (%)	27 (3.2)	9 (2.7)	5 (1.7)	13 (6.3)
Other, n (%)	10 (1.2)	5 (1.5)	3 (1.0)	2 (1.0)

^a^Includes individuals known to be HIV positive at the time of their early syphilis diagnosis

^b^The critical period for increased likelihood of transmission is defined as 12 months for and HIV diagnosis, and 3, 6, and 12 months for a primary, secondary, and early latent syphilis diagnosis, respectively

^c^Includes reports of injection drug use or any method of consumption of the following drugs—cocaine, heroin, and/or methamphetamines

On average, individuals were 27 years old [interquartile range (IQR) 11.00], 79.1% were Black (n = 507), and 5.0% were Latino (n = 32). The median number of sex partners reported was 2 (IQR 50.00), 7.2% (n = 46) of individuals reported engagement in commercial sex work, 8.7% (n = 56) reported any drug use, 53.8% (n = 345) reported anonymous sex, and 95.0% (n = 609) reported sex without a condom. The 641 individuals nominated 979 venues, of which 85.4% (n = 836) were nominations of venues reported by more than one MSM and were included in this analysis. The median number of venue reports per individual was 1 (IQR 7.00).

*Objective one*: *characterize the connectivity of sex partner meeting places to assess venue co-affiliation network structure and examine syphilis and HIV transmission overlap.*

In the venue co-affiliation network of venues from 2009 to 2017, there were 57 unique venues and among these, 7.0% (n = 4) were classified as syphilis venues, 21.1% (n = 12) as HIV venues, and 71.9% (n = 41) as co-diagnosed venues. A majority of the venues were online venues (45.6%, n = 26) followed by bars or clubs (15.8%, n = 9). The remaining venues were streets, parks, or neighborhoods (10.5%, n = 6), markets or malls (8.8%, n = 5), or other (5.3%, n = 3). Almost half of all venues (42.1%, n = 24) had at least one report of commercial sex work, and almost half of all venues (42.1%, n = 24) had at least one report of drug use. The percent of reports of commercial sex work at each venue ranged from 0 to 100% (median 0%, IQR 13.33%). The percent of reports of any drug use at each venue ranged from 0 to 50% (median 0%, IQR 12.50%) A total of 94.7% (n = 54) of venues had at least one report of anonymous sex, and 100.0% (n = 57) of venues had at least one report of condomless sex. The percent of reports of anonymous sex at each venue ranged from 0 to 100% (median 60%, IQR 21.42%), and the percent of reports of condomless sex at each venue ranged from 50 to 100% (median 100%, IQR 5.41%).

Overall, the median degree centrality was 4 (IQR 54.00). Four venues, Facebook, Adam4Adam, Jack’d, and Instagram, represented 51.6% of all unique venue reports. All four are online venues and were classified as co-diagnosed venues. These four venues had the highest degree centralities of 152, 101, 64, and 54, respectively. These four venues also had the highest eigenvector centralities of 1.00, 0.50, 0.27, and 0.22, respectively.

Of the 57 unique venues, 86.0% (n = 49) were connected in one main network component of which 6.1% of venues (n = 3) were syphilis venues, 20.4% (n = 10) were HIV venues, and 73.5% (n = 36) were co-diagnosed venues. To assess transmission overlap, connectivity of single diagnosis venues (i.e. syphilis only and HIV only venues) and co-diagnoses venues were described, and centrality measures were compared between single and co-diagnosis venues. Among single diagnosis venues, 69.2% (n = 9) were directly connected to at least one co-diagnosis venue, 7.7% (n = 1) were indirectly connected (i.e. connected through another single-diagnosis venue) to a co-diagnosis venue, and 23.1% (n = 3) were not connected to any other venue (i.e. an isolate). Among co-diagnosis venues, 100% (n = 36), were directly connected to another co-diagnosis venue, and 2 co-diagnosed venues formed the only dyad (i.e. pair of venues disconnected from the main network component). 33% (n = 12) of co-diagnosis venues were directly connected to at least 1 single diagnosis venue, 52.8% (n = 19) were indirectly connected to a single diagnosis venue, and 8.3% (n = 3) were isolates.

**Fig. 1:**
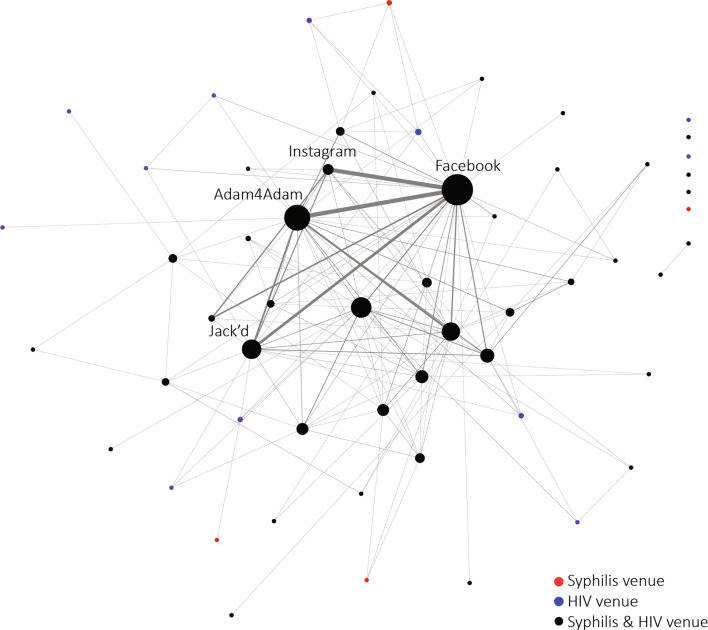
Co-affiliation network of sex partner meeting places (n = 57) reported more than once among men who has sex with men (MSM) with an early syphilis and/or a new HIV diagnosis (n = 641), Baltimore City, 2009–2017. Venues are linked if they have at least one shared case. The color of the node reflects the venue level diagnosis: red is syphilis only nominations, blue is HIV only nominations, and black is co-diagnosed nominations. The size of the node reflects the degree centrality (i.e. a larger node represents more venue connections). The width of the line indicates tie strength (i.e. a wider tie reflects more shared cases between venues) (Color figure online)

Single diagnosis venues had significantly lower median degree centralities compared to co-diagnosed venues [3.5 (IQR 3.00), 5.0 (IQR 53.00), Mann–Whitney Fishers p < 0.01, respectively]. The median eigenvector centrality was not significantly different between the two groups [0.00 (IQR 0.01), 0.01 (IQR 0.03), Mann–Whitney Fishers p = 0.15, respectively, Fig. 1].

*Objective two*: *describe temporal changes in the co-affiliation network structure from 2010 to 2014.*

Over the 5-year period from 2010 to 2014 when early syphilis cases increased 40% among MSM, 408 MSM nominated 54 unique venues.

The median degree centrality increased 33% from 3 (IQR 4.00) in 2010 to 4 (IQR 13.00) in 2014 (Mann–Whitney Fishers p = 0.49), and the median eigenvector centrality decreased 78% from 0.14 (IQR 0.22) in 2010 to 0.03 (IQR 0.09) in 2014 (Mann–Whitney Fishers p = 0.09, Table 2). There were no differences in age, race, ethnicity, or diagnosis comparing 2010 to 2014 (*data not shown*).

**Table 2:** Measures of co-affiliation network structures (i.e. total number of venues, degree centrality, eigenvector degree centrality) and density (i.e. connectedness, compactness) among sex partner meeting places (n = 32) reported more than once among gay, bisexual and other men who have sex with men (MSM) diagnosed with early syphilis and/or a new HIV diagnosis (n = 408), during a time period of increases in early syphilis cases among MSM, Baltimore City, 2010–2014

Year	Number of venues reported by > 1 MSM	Degree centrality of venues	Eigenvector degree centrality of venues	Connectedness of network	Compactness of network
	n	Median (IQR)	Median (IQR)	Value	Value
2010	13	3 (4.00)	0.14 (0.22)	0.47	0.36
2011	11	2 (6.00)	0.07 (0.27)	0.51	0.34
2012	13	2 (4.00)	0.04 (0.13)	0.58	0.38
2013	18	3 (7.00)	0.03 (0.07)	0.89	0.52
2014	22	4 (13.00)	0.03 (0.09)	0.74	0.46
% Change 2010–2014	+ 69	+ 33	− 79	+ 57	+ 28

Networks were created by linking venues that shared at least one case of early syphilis and/or HIV

**Fig. 2:**
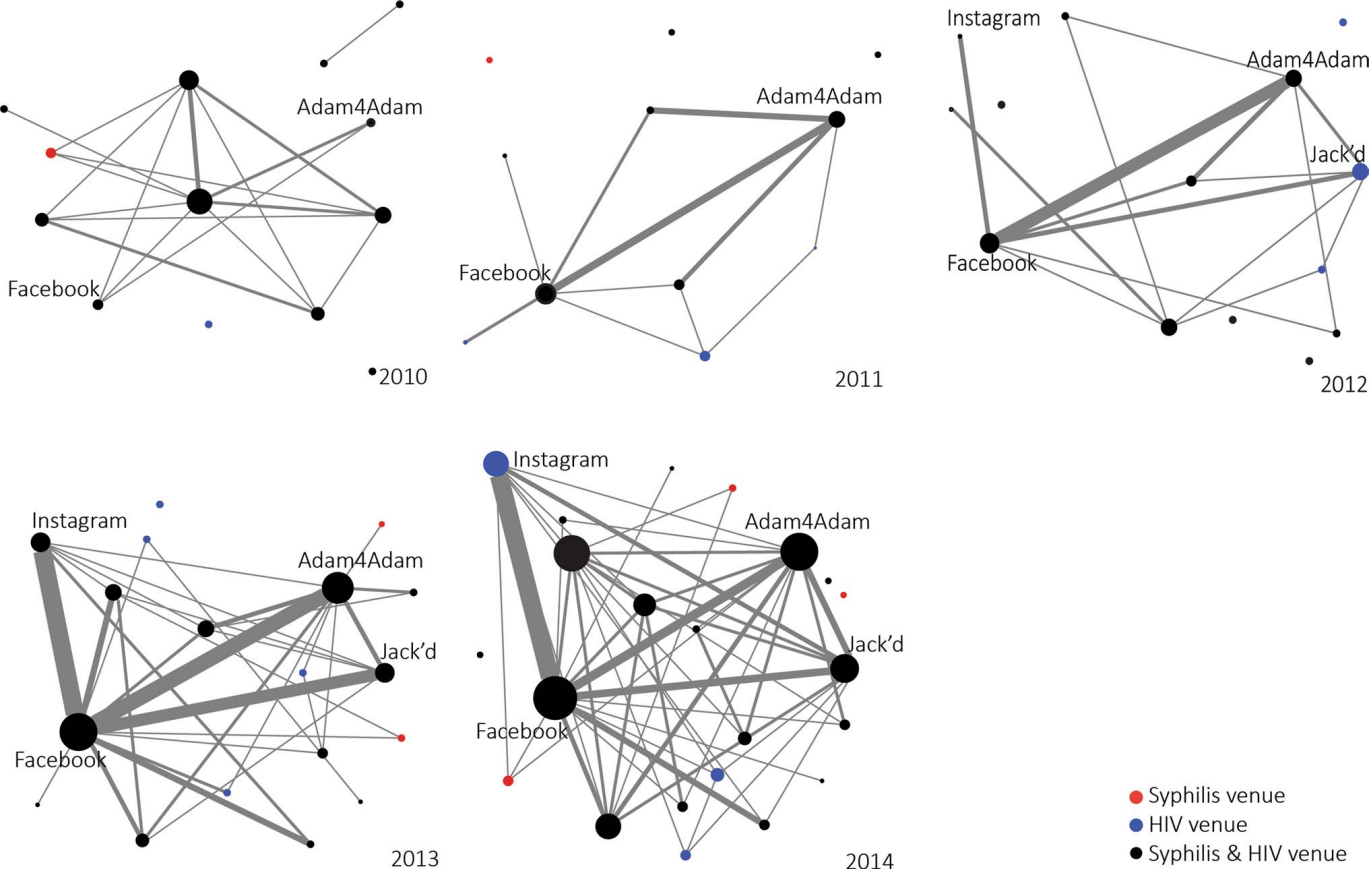
Co-affiliation networks by year of sex partner meeting places (n = 54) reported more than once among men who have sex with men (MSM) diagnosed with an early syphilis and/or a new HIV diagnosis during a time period of increases in early syphilis cases among MSM, Baltimore City, 2010–2014 (n = 408). Venues are linked if they have at least one shared case. The color of the node reflects the venue level diagnosis: red is syphilis only nominations, blue is HIV only nominations, and black is co-diagnosed nominations. The size of the node reflects the degree centrality (i.e. a larger node represents more venue connections). The width of the line indicates tie strength (i.e. a wider tie reflects more shared cases between venues (Color figure online)

Of the four venues with the highest degree centralities and eigenvector centralities across the 5-year period, Facebook and Adam4Adam were nominated all 5 years, and Instagram and Jack’d were first nominated in 2012. The degree centrality of these venues and tie strength between these venues increased annually from 2010 to 2014 (*data depicted visually in* Fig. 2).

The total number of venues nominated by more than one MSM increased 69.2% (n = 13 to 22, Fig. 2). The proportion of co-diagnosed venues decreased from 85% in 2010 to 73% in 2014. Comparing 2010 to 2014, the connectedness (i.e. proportion of venues connected in the main component) increased 57% from 0.47 to 0.74, and compactness (i.e. connectedness, weighted inversely by the length of the ties) increased 28% from 0.36 to 0.46 (Table 2).

## Discussion

The overall goal of this study was to inform innovative, data-driven public health prevention and control strategies for reducing STIs and HIV. The focus was on sex partner meeting places as an important means to access priority populations and their networks to reduce the transmission of syphilis and HIV. The approach utilizing venue co-affiliation analyses inherently recognizes that sex partner meeting places form a network of venues, i.e. individuals attend more than one venue, which may yield important information for the optimization of public health control strategies [22–25, 27].

Our approach is novel in that the design sought to address the syndemic of syphilis and HIV instead of treating each disease separately as is often the approach [37]. In addition, we applied a novel methodology, venue co-affiliation network analyses, and the findings have important implications for public health programs considering how best to allocate resources optimally for syphilis and HIV control [17, 26, 27].

The median age of the study population of MSM was 27 years (IQR 11.00), and 79.1% were Black. These demographics are notable because in Baltimore City, as well as other cities across the U.S., young Black MSM are a population that has continued to experience a high incidence and, in some places, an increasing incidence of syphilis and HIV [1–3]. Targeted and tailored control strategies are desperately needed for this population to reverse the epidemic.

The venue co-affiliation networks of early syphilis and/or newly HIV diagnosed MSM suggest a high degree of overlap in the potential transmission of both syphilis and HIV transmission networks. Approximately 72% of venues were co-diagnosed venues. When comparing the median degree centrality of the venues, single diagnosed venues had a lower median degree than co-diagnosed venues demonstrating that these co-diagnosed venues are overall more central indicating that they have more shared patrons with other venues than single diagnosis venues. In addition, almost half of the venues were reported by individuals also reporting high sexual risk behaviors such as commercial sex work (41%) and drug use (41%). Four venues, Facebook, Adam4Adam, Jack’d, and Instagram, represented 51.6% of all unique venue reports and were all online, co-diagnosed venues. These venues also had the highest degree centrality and eigenvector centralities. These network measures suggest that these four venues had the most nominations and shared the most ties with other venues in the network, i.e. they were connected to the most venues that also had a high number of other venue ties. Other venue-based studies have identified a similar pattern of a dense core of venues, although most have identified a core with both physical and online venues [23, 24].

The four online core venues we identified may represent the optimal set for tailored interventions. Interventions, for example, could include offering HIV/STI testing, sexual risk reduction behavioral interventions and/or PrEP through messaging on the apps. The data also suggest, however, that an intervention targeting each of the four venues may be redundant, and optimal interventions may be able to target one or two of the highly central venues and reach a majority of the venues and individuals. The broader network of venues also suggests that it may be important to additionally target venues that are less closely tied to this highly connected set of four venues.

In addition, it is notable that the majority of nominated sex partner meeting places (53.1%) were online venues. Our prior work showed increasing trends in nominations of online sex partner meeting places, but also suggested that physical places remained important and highly connected to online spaces [22, 34]. Our current work suggests that sex partner meeting venues are dynamic and for MSM, may be increasingly online. In part this may also be due to other social forces. In Baltimore City, for example, a number of bars and clubs specifically tailored to MSM have closed in the past 5 years. This trend may in part be due to an increasing social acceptance of MSM with, for example, the legalization of same sex marriages in Maryland in 2013. It may also be due to the increasing availability and use of online venues particularly among youth who are increasingly digital natives. The dynamic nature suggests that patterns of sex partner meeting places are changing, and local health departments need to modify current approaches and/or develop new strategies and interventions that include online approaches.

Our analysis of temporal changes of the venue connectivity over a 5-year period of increasing early syphilis cases among MSM suggests increasing nomination, density and connectivity of the co-affiliation network structure over the time period. The network structure changes over time may also suggest higher transmission rates and increased difficulty of interrupting network transmission through fragmentation. The findings suggest a 69.2% increase in the number of venues that were reported by at least 2 MSM. The proportion of venues connected in the main component as measured by connectedness increased 57% over the 5 years, suggesting an increasing density of venue connection over time and potentially increasing transmission potential over time. The increase in compactness of 28% over the 5 years is also crucial because it suggests that the paths between venues are shorter and therefore, transmission may be more efficient.

These results may reflect either an increasing number or use of venues for MSM to meet sex partners, or alternatively a condensing of venues for MSM to meet sex partners, therefore increasing the likelihood that a venue is nominated by more than one individual. These two scenarios—increasing number or use versus condensing of venues—are very different phenomena and each has different implications for transmission and intervention. The former scenario may result in greater challenges in zeroing in on the transmission sources. The latter scenario in contrast, may present fewer challenges with fewer venues to choose from.

There are important limitations to this study. Data were limited to venues reported by MSM with an early syphilis and/or new HIV diagnosis. The men reporting venues may be different than those not reporting venues, although our statistical testing did not suggest differences. In addition, the data reported on venues may be subject to recall bias and may not represent all venues MSM frequented to meet sex partners in the past 12 months. Also, because co-affiliation network analysis is a tool to identify opportunities for social and sexual connection, these data are not necessarily a reflection of where direct connections occurred or where the infection was acquired. Missing information in the surveillance data did not allow for a thorough examination of individual risk information such as drug use, commercial sex work, unprotected sex, and co-infection, which if incorporated may have helped to further elucidate the transmission potential of venues and assist in tailoring control strategies. In addition, we did not interview non-infected individuals to assess how their networks may differ. Another limitation was that we simplified the analyses by comparing single vs. co-diagnosis venues and this may have minimized some nuances related to the connectivity of the venues. Finally, there are different interpretations of what it means to be central in an affiliation network, and a related challenging aspect is that we assume one-mode relationships of patron co-memberships without having important information about the pattern of affiliation ties [38].

In summary, this approach is innovative as it harnesses the potential power of routinely collected public health surveillance data, may be more reliable than traditional contact tracing sexual network data, and importantly, focuses on the inherent networks of sex partner meeting places to identify transmission networks and overlapping transmission networks of syphilis and/or HIV [26]. Leveraging this information may help to increase the efficiency of syphilis and HIV prevention and control strategies and maximize the use of public health funds. The highly nominated places and highly connected venues (i.e. high degree centrality and eigenvector centrality) may help direct local programs to places for prioritized outreach and linkage to care to reduce ongoing transmission, but the less nominated and connected venues may also be important since interventions would reach them in a time lagged way or not at all if they are disconnected. Future research should seek to develop, test and evaluate control strategies based on these findings to determine whether these control strategies result in the identification of more syphilis and HIV positives, specifically those individuals who are infectious, and whether ultimately the strategies result in future declines in syphilis and HIV transmission.
